# Localized atrial fibrillation within the inferior sinus venosa treated by isolation: Could it be a new target of ablation for persistent atrial fibrillation?

**DOI:** 10.1016/j.hrcr.2025.05.003

**Published:** 2025-05-08

**Authors:** Yuya Suzuki, Tomomi Akita, Kaoru Takami, Takamichi Ishihata, Akihiro Yoshida

**Affiliations:** 1 Department of Cardiology, Kita-Harima Medical Center, Ono, Japan; 2 Department of Clinical Engineering, Kita-Harima Medical Center, Ono, Japan

**Keywords:** Inferior sinus venosa Atrial fibrillation, Right atrium, Trigger, Maintenance

**Key Teaching Points**

• The inferior part of sinus venosa (inferior SV) was considered to be rare but important as an atrial fibrillation (AF) substrate.
• The inferior SV was considered to be rare but important as an AF trigger.
• Adjunctive right atrial (RA) ablation, including the inferior SV, should be considered for the pulmonary vein isolation–resistant cases of persistent AF with RA enlargement and atrial premature contraction originating from the inferior and posterior part of RA.

## Introduction

The pulmonary veins (PVs) are the major source of ectopic beats responsible for the occurrence and maintenance of atrial fibrillation (AF).^1^ Although PV isolation (PVI) is the standard ablation strategy for patients with AF, several adjunctive strategies are required for patients with AF who are refractory to PVI.^2^ Multiple trials of substrate modification for AF rotors, including the right atrium (RA), have been conducted in patients with persistent AF after PVIs. The superior part of the sinus venosa (SV) has been reported to be a rare source of atrial tachycardia during AF ablation.^3^ We report a rare case in which AF was spontaneously initiated and maintained only within the inferior SV and successfully ablated by the isolation of this area.

## Case report

**Figure 1:**
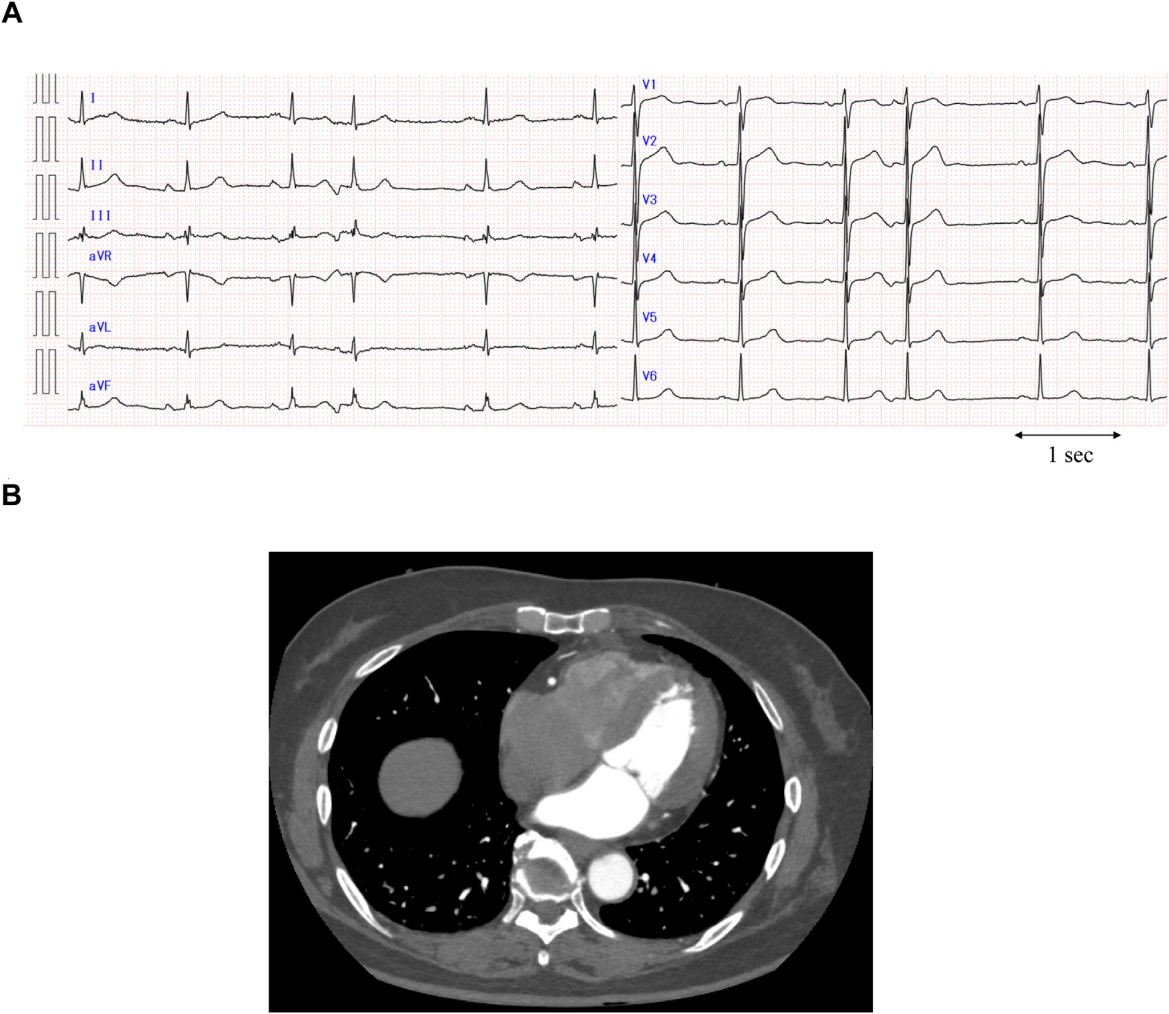
Twelve-lead electrocardiogram and computed tomography image before atrial fibrillation ablation. **A:** A 12-lead electrocardiogram of sinus rhythm and atrial premature contraction in a patient with recurrent atrial fibrillation after the first session of catheter ablation. atrial premature contraction exhibited negative P waves in the inferior leads, isoelectric P waves in lead I, and positive P waves in lead V1. **B:** Axial view of the cardiac computed tomography before ablation.

A 69-year-old woman was admitted to our hospital with complaints of palpitations. Baseline 12-lead electrocardiography revealed AF and frequent atrial premature contractions (APCs). APC exhibited negative P waves in the inferior leads, isoelectric P waves in lead I, and positive P waves in lead V1 (Figure 1A). Mild tricuspid regurgitation was detected but the left ventricular ejection fraction and left atrial dimension were found to be normal using transthoracic echocardiography. RA enlargement was observed in cardiac computed tomography (Figure 1B).

The patient had previously undergone PVI and cavotricuspid isthmus ablation for paroxysmal AF, but AF recurred and was refractory to medication, which led to performing a second ablation.

**Figure 2:**
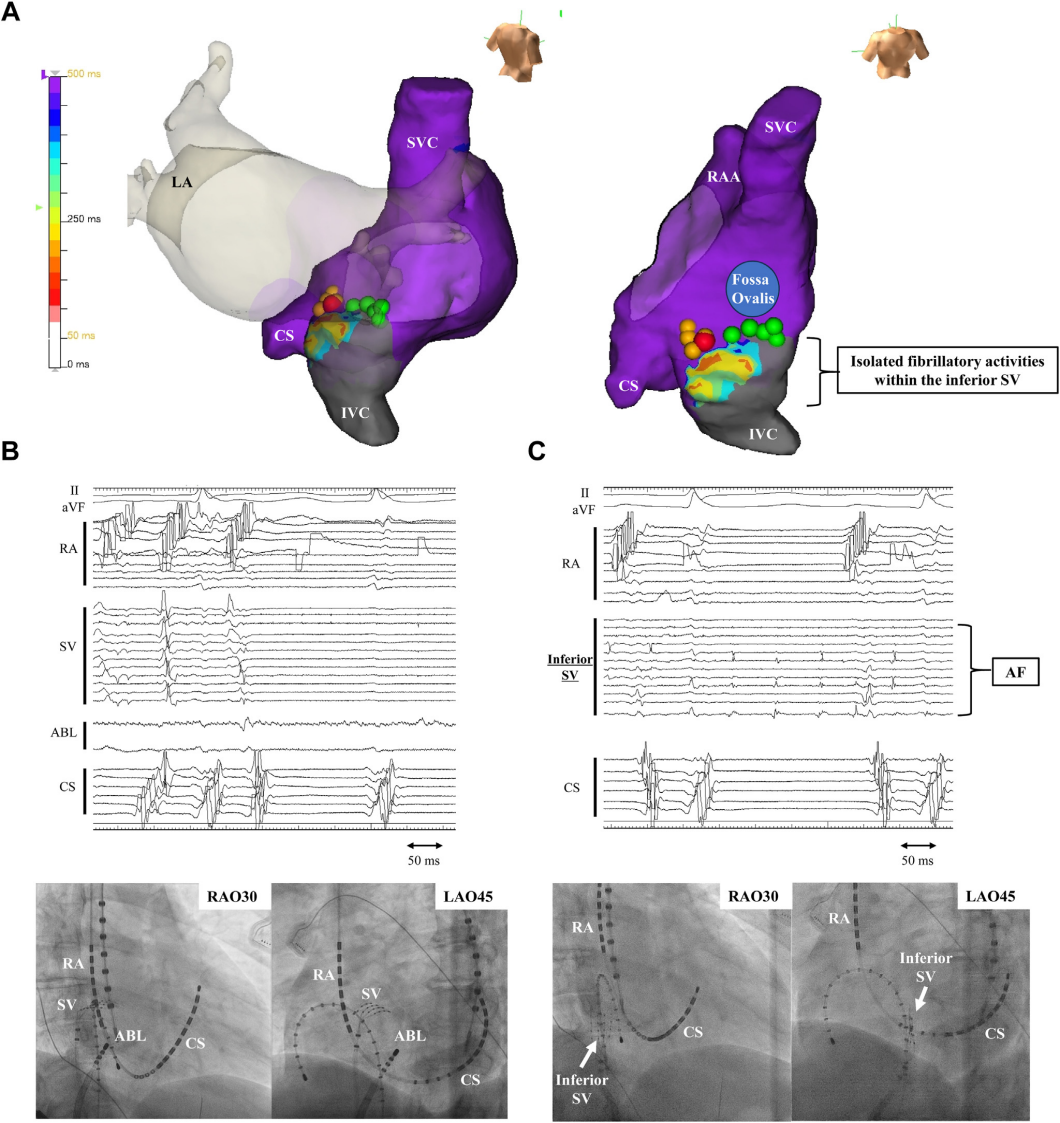
Three-dimensional mapping and intracardiac electrogram expressing that localized atrial fibrillation (AF) was observed only within the inferior sinus venosa (SV). **A:** Complex fractionated electrogram mean diagnostic landmark mapping (EnSite system) of RA showing that localized AF was observed only within the inferior SV. Complex fractionated electrogram mean was the average time interval between consecutive deflections over 5 seconds. A mean cycle length of < 500 ms was considered to represent AF, as the sinus rate was 60 beats per minute. Localized AF was observed only within the inferior SV, whereas other areas of the RA showed sinus rhythm. Green tag, first ablation point (fossa ovalis); yellow tag, second ablation point (upper side of the inferior SV); red tag, AF termination point. **B:** Intracardiac electrogram. The ablation of the upper part of the inferior SV (Figure 1A, *red tag*) converted AF to sinus rhythm. **C:** Intracardiac electrogram. Localized AF was maintained only within the inferior SV, whereas the other areas of the atrium showed sinus rhythm. ABL = ablation;; CS = coronary sinus; IVC = inferior vena cava; LA = left atrium; RA = right atrium; SVC = superior vena cava.

Electroanatomic mapping using the EnSite system (Abbott Laboratories, Abbott Park, IL) and a high-resolution mapping catheter (Advisor HD Grid, Abbott Laboratories) was conducted. The right PV was reisolated because of reconnection (Supplemental Figure 1). When non-PV triggers were induced by administering a continuous infusion of isoproterenol (0.1 μg/kg/min) without atrial burst pacing, immediate recurrence of incessant AF was observed, and the initial APC was identified using a high-resolution mapping catheter. Given that non-PV triggers originated from the atrial septum near the fossa ovalis, we performed mapping of the atrial septum using a catheter positioned on the RA and LA sides. Because the APC was recorded earlier on the RA side than on the LA side, ablation was attempted from the RA side (Figure 2A, first ablation point indicated by green tags). However, AF recurred immediately.

We added further mapping of non-PV triggers that revealed that AF was initiated from the inferior part of SV, which was located above the inferior vena cava (IVC), below the fossa ovalis, and between the eustachian ridge and posterior boundary (Supplemental Figure 2).

**Figure 3:**
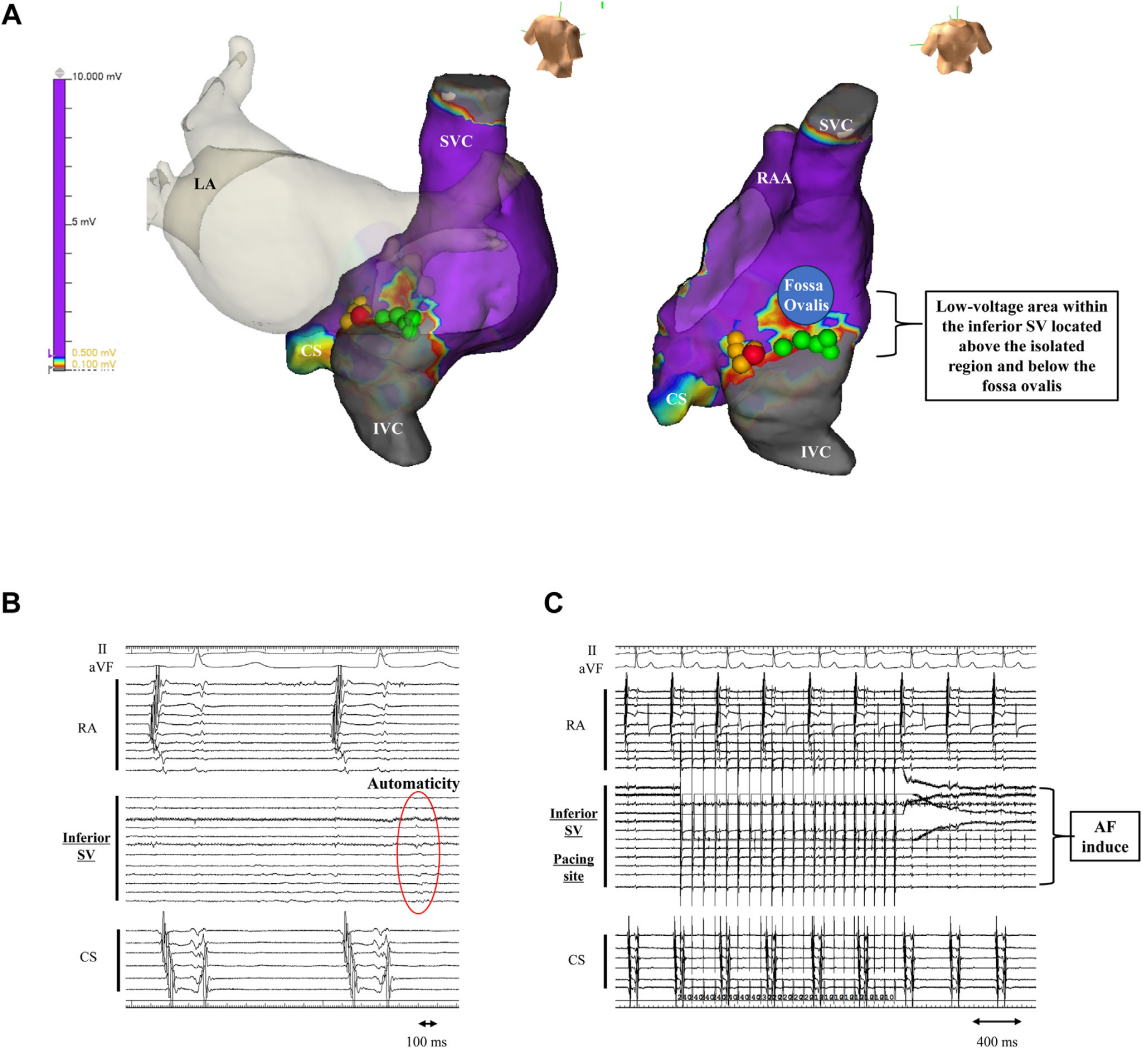
Low-voltage area and arrhythmogenicity of the inferior sinus venosa (SV). **A:** High-density voltage mapping of the right atrium (RA) during sinus rhythm. The low-voltage area in the RA during sinus rhythm was defined as a bipolar voltage of <0.5 mV. **B:** Intracardiac electrogram. Automatic electrical activity (*red circle*) inside the isolated inferior SV was observed. **C:** Intracardiac electrogram. Rapid atrial pacing (50 ms/30 mA) at the isolated inferior SV induced localized AF. The colored tags have the same settings as in Figure 1. ABL = ablation; CS = coronary sinus; IVC = inferior vena cava; LA = left atrium; RA = right atrium; SVC = superior vena cava.

We ablated the earliest atrial activation sites of non-PV triggers located at the upper part of the inferior SV (Figure 2A, second ablation point; yellow tags), and AF returned to sinus rhythm (Figure 2B, AF termination site; *red tag*). During sinus rhythm, re-mapping of the RA interestingly showed that localized AF incessantly appeared only inside the isolated area of inferior SV, whereas sinus rhythm was maintained in the outside area of the RA (Figure 2A and 2C). After spontaneous termination of the localized AF, the isolated area of the inferior SV showed localized automatic electrical activity (Figure 3B). A low-voltage area existed above the isolated inferior SV below the fossa ovalis; however, no abnormalities were observed in the other RA area (Figure 3A). After ablation, any atrial tachycardia and AF were not induced by isoproterenol infusion and atrial burst pacing at the 3 sites (the distal coronary sinus, proximal coronary sinus, and superior RA) from 300 ms to 2:1 atrial capture. Rapid atrial pacing from the isolated area of the inferior SV induced localized AF only inside the limited area (Figure 3C). After the discharge, there was no AF recurrence, and the patient did not experience any palpitations. This case highlights the inferior SV as one of the important sites among non-PV triggers and AF drivers. The inferior SV might need to be considered as the target site for refractory AF.

## Discussion

For the first time, we detected localized AF maintained only within the inferior SV and successfully ablated by isolating this area. The inferior SV was considered to be important as an AF substrate.

The SV is an area with a smooth inner wall and is located within the posterior RA between the superior and IVC and between the RA septum and the crista terminalis.

Takami and colleagues^4^ showed that the lower part of the posterior boundary located in the lateral SV was a fixed conduction block disturbing transverse conduction until the IVC, and this functional block extended upward from the fixed conduction block depending on the pacing rate and site. Moreover, Nakagawa and colleagues^5^ reported that the eustachian ridge formed the fixed conduction block between the IVC and the coronary sinus ostium. In this case, we speculated that the first ablation region of the RA septum for non-PV triggers formed a block to the RA posterior side, and the second ablation region formed a block to the eustachian ridge, resulting in the isolation of the inferior SV by the 4 blocks of the innate posterior boundary (lateral side) and the eustachian ridge (septal side) and the ablation sites below the fossa ovalis (upper side) and IVC (lower side).

Although the PVs and LA are generally considered important AF substrates, several reports have shown that the SV has been related to AF arrhythmogenicity. A swine model histologic study showed that SV consisted of muscle bundles with a nonuniform fiber direction and surrounded by collagen tissue.^6^ SV is a rare non-PV trigger (4.4% of all non-PV triggered cases)^7^ and a very rare origin of focal AT (0.82% of all ATs during AF ablation).^3^ Moreover, AF rotors were occasionally observed in the RA but rarely in the SV. In contrast, the ablation of AF rotors within the SV led to a frequency reduction and promoted AF termination.^8^^,^^9^ Furthermore, the RA ganglionated plexi were frequently observed in the inferoposterior of the RA, and the inferior SV may be associated with the RA ganglionated plexi.^10^

In this case, we noted that localized AF was maintained only within the inferior SV, whereas sinus rhythm was observed in areas other than the isolated inferior SV, suggesting that the inferior SV is a crucial area as an AF trigger and substrate.

In a recent study, adjunctive RA ablation increased the success rate of a single ablation in patients with persistent AF and RA enlargement.^11^ In this case, it was assumed that additional RA ablation was required owing to RA enlargement and APCs originating from the inferior SV. In the procedure, the electrical remodeling of the RA (especially the inferior SV) was observed by the voltage mapping during sinus rhythm. We considered adjunctive RA ablation, including inferior SV ablation, in PVI-resistant cases of persistent AF with RA enlargement.

Further studies are needed to confirm whether the inferior SV could be a new target of AF ablation for patients with RA dilatation.

## Conclusion

We reported a rare case in which localized AF was maintained only within the inferior SV and was successfully ablated by isolating this area. The inferior SV was considered to be an important AF substrate.

## Disclosures

The authors have no conflicts of interest to disclose.
