# Supplementary material for: Localized atrial fibrillation within the inferior sinus venosa treated by isolation: Could it be a new target of ablation for persistent atrial fibrillation?
Source: HeartRhythm Case Rep. 2025 May 8;11(7):684–8. doi: 10.1016/j.hrcr.2025.05.003 (PMC12432982; doi:10.1016/j.hrcr.2025.05.003)
Supplement: Supplementary Material [file mmc1.pptx]

## Slide 1
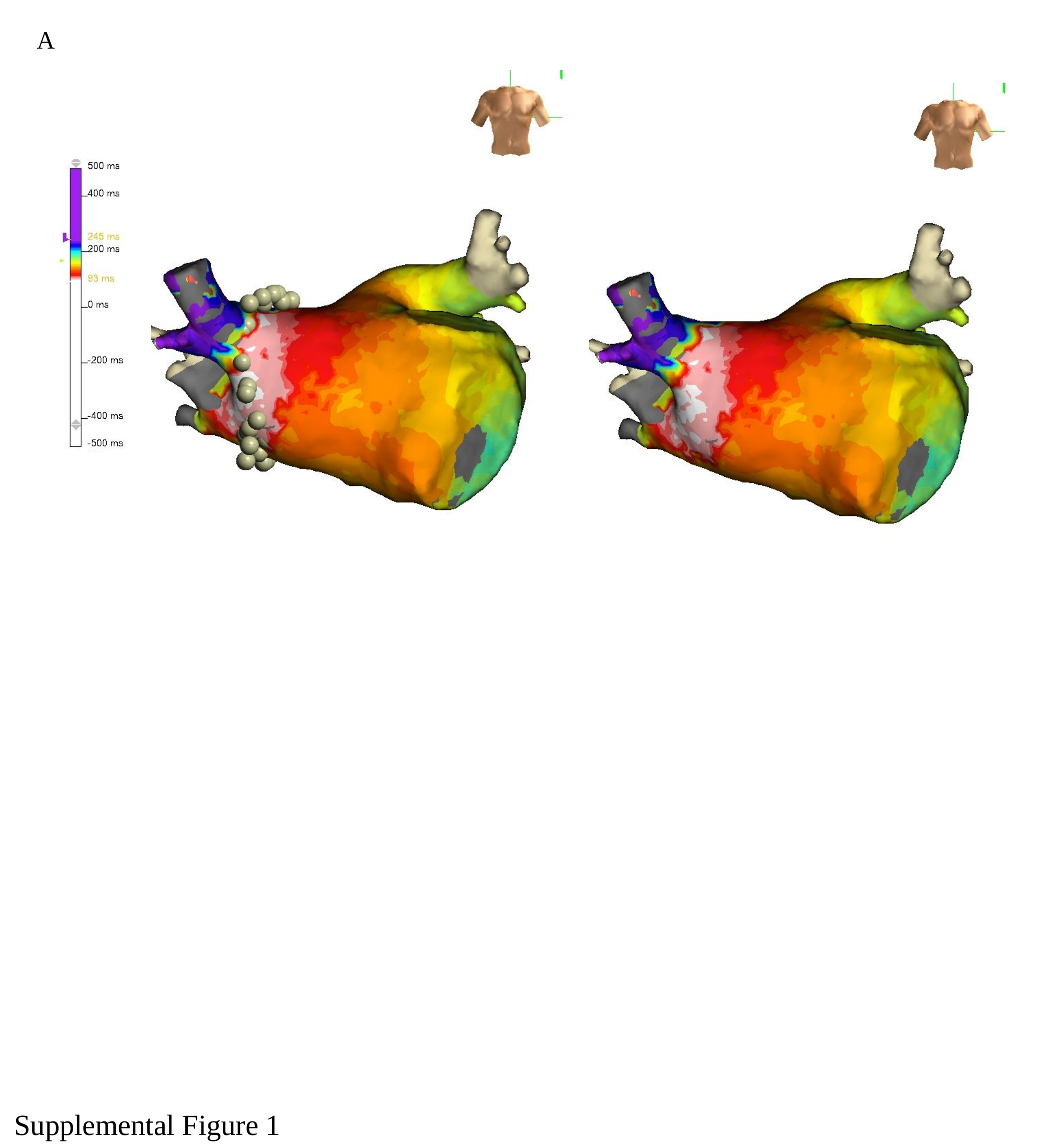

A
Supplemental Figure 1

## Slide 2
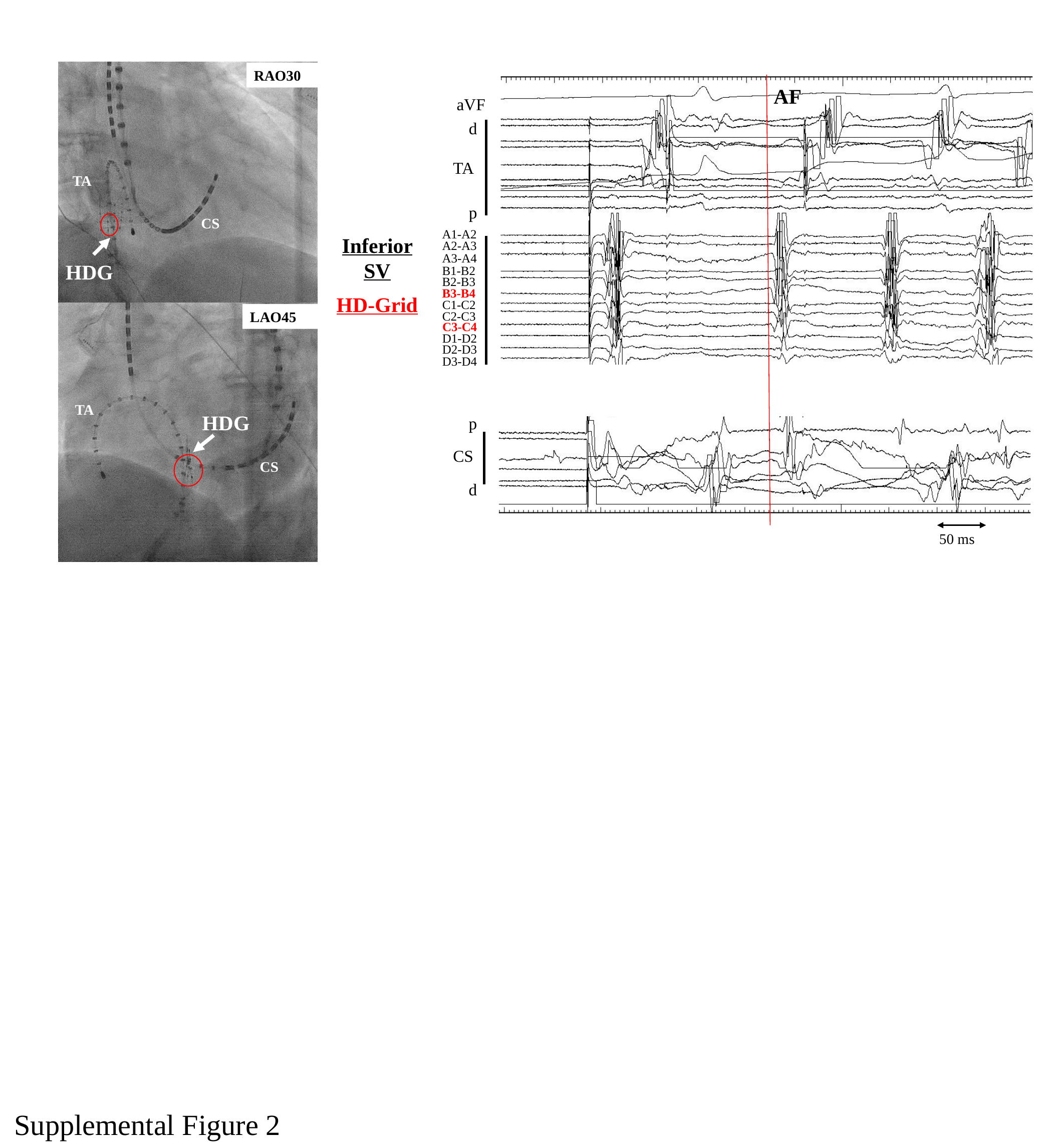

RAO30
AF
aVF
d
TA
TA
p
CS
A1-A2
Inferior
SV
A2-A3
A3-A4
HDG
B1-B2
B2-B3
B3-B4
HD-Grid
C1-C2
LAO45
C2-C3
C3-C4
D1-D2
D2-D3
D3-D4
TA
HDG
p
CS
CS
d
50 ms
Supplemental Figure 2
