# Supplementary material for: Localized atrial fibrillation within the inferior sinus venosa treated by isolation: Could it be a new target of ablation for persistent atrial fibrillation?
Source: HeartRhythm Case Rep. 2025 May 8;11(7):684–8. doi: 10.1016/j.hrcr.2025.05.003 (PMC12432982; doi:10.1016/j.hrcr.2025.05.003)
Supplement: Supplementary Figures 1 and 2 [file mmc2.docx]

**Supplemental Figure 1. Three-dimensional mapping regarding the ablation of reconnected right PV.**

**A**, Three-dimensional mapping of the activation mapping and ablation point of the reconnected right PV; tag = ablation point.

**Supplemental Figure 2. Intracardiac electrograms during non–pulmonary vein (non-PV) trigger mapping.**

The earliest activation of the APC-triggered atrial fibrillation (AF) originated from the inferior sinus venosa, where the B3–B4 and C3–C4 electrodes of the Advisor™ HD Grid catheter were positioned.
